# Characteristics of Carbonatogenic Bacteria and Their Role in Enhancing the Stability of Biocrusts in Tropical Coral Islands

**DOI:** 10.3390/microorganisms13030523

**Published:** 2025-02-27

**Authors:** Qiqi Chen, Lin Wang, Jie Li, Qiqi Li, Hongfei Su, Zhimao Mai

**Affiliations:** 1 School of Resources, Environment and Materials, School of Marine Sciences, Guangxi University, Nanning 530004, China; 2 CAS Key Laboratory of Tropical Marine Bio-Resources and Ecology, South China Sea Institute of Oceanology, Chinese Academy of Sciences, Guangzhou 510301, China

**Keywords:** biocrusts, carbonic anhydrase, shear strength, sand consolidation

## Abstract

Soil erosion is a serious environmental problem that leads to land degradation and ecological imbalance, thereby eliciting extensive and profound worldwide concern. Biological soil crusts (biocrusts) play a crucial role in soil stabilization; however, the underlying microbial enzymatic mechanisms remain poorly understood. The present study aimed to characterize carbonatogenic bacteria and investigate the role of their carbonic anhydrase-induced carbonate crystals in promoting soil shear strength within biocrusts. The results demonstrated a significant increase in the activity of carbonic anhydrase during biocrust formation and development (*p* < 0.05). A total of 35 strains exhibiting carbonic anhydrase activity were isolated from biocrusts, belonging to Actinomycetota, Bacillota, Pseudomonadota and Cyanobacteriota. The subsequent investigation revealed a positive correlation between the carbonic anhydrase activities of the strains and the shear strength during sand consolidation. Specifically, strain SCSIO19859, a type of cyanophyta, exhibited the highest carbonic anhydrase activity, of 1.50 U/mL. It produced 0.70 g/day of calcium carbonate and demonstrated a shear strength that was 6.09 times greater than that of the control group after sand consolidation for seven days of incubation under optimal conditions. X-ray diffraction and scanning electron microscope analysis revealed that SCSIO19859 produced calcite and vaterite carbonates, which significantly increased the shear strength of the sand grains (*p* < 0.05). This study provides evidence for the ecological function of biocrusts in promoting soil erosion resistance from the perspective of carbonatogenic bacteria-derived carbonic anhydrase. The functional strains with carbonic anhydrase obtained from this study have significant potential applications in enhancing soil erosion resistance.

## 1. Introduction

Soil erosion, resulting from both natural factors (wind or water erosion) and human activities that remove soil from the surface, is a widespread form of land degradation, affecting 84% of degraded lands [1,2]. Soil erosion leads to several severe issues, including the loss of soil organic carbon [3], a reduction in biodiversity [4] and an increase in the occurrence of natural disasters such as landslides and dust storms [5,6]. Globally, wind and water erosion affect more than 1.5 billion hectares of land, whereas in China, the area of water and soil loss exceeds 26 million hectares, primarily occurring in desert regions, plateaus, grasslands and islands [7,8,9]. Particularly on tropical coral islands, nutrient availability is limited within the coral sand matrix, resulting in sparse vegetation cover. Moreover, these coral islands are exposed to extreme environmental conditions such as intense rainfall events and frequent storms that significantly increase the vulnerability of loose coral sand to erosion [10]. Hence, it is imperative to implement measures for mitigating soil erosion in these regions.

Biological soil crusts (biocrusts) are intricate composites formed through the adhesion of microorganisms such as bacteria, fungi and algae (including cyanobacteria and algae), as well as cryptogamic plants such as mosses, along with their mycelia and secretions, to soil particles [11]. These pioneer organisms can thrive in extreme environments characterized by high temperatures, intense light exposure and limited water availability; therefore, they are vital for restoring desert ecosystems [12]. Biocrusts are essential in providing soil ecosystem services, including accelerating soil respiration, enhancing photosynthesis, improving fertility, regulating water flow and facilitating weathering processes [13,14,15,16]. More importantly, biocrusts can mitigate wind and water erosion effectively, thereby enhancing soil stability [17]. Consequently, they have been extensively utilized in desert and plateau regions to ameliorate soil stability and diminish erosion [18,19]. The involvement of microorganisms is crucial in the process of sand stabilization in biocrusts. The intertwining of microbial hyphae with soil particles results in the formation of aggregates that increase soil stability [20]. Moreover, microbial secretion of extracellular polymeric substances contributes to promoting soil structural stability by adhering to soil particles [21,22]. Additionally, these substances contain diverse active components, including microbial enzymes [23]. However, few studies have investigated the mechanisms through which microbial enzymes increase the erosion resistance of biocrusts.

Carbonic anhydrase is a zinc-containing metalloenzyme that can catalyze the hydration reaction of carbon dioxide, thereby facilitating the formation of carbonates. This enzyme is widely distributed among living organisms and is characterized by its remarkable catalytic efficiency [24]. Current research suggests that carbonic anhydrase has extensive applications in soil stabilization, enhancing the performance of concrete materials and restoring damaged buildings and cultural relics [25,26]. The findings from our previous research indicate a positive correlation between erosion resistance and microbial carbonic anhydrase activity in biocrusts [27]. The result indicated that biocrusts may harbor carbonatogenic bacteria with elevated carbonic anhydrase activity, which could play a pivotal role in promoting sand stabilization. The further isolation and screening of carbonic anhydrase strains from biocrusts, along with the elucidation of the role of carbonates in sand consolidation, hold significant importance for future applications.

Therefore, cyanobacterial biocrusts at different developmental stages were selected. The purposes of this study were to (1) elucidate the variations in carbonic anhydrase activity during biocrust formation and development, (2) isolate microbial strains with carbonic anhydrase activity and (3) identify the characteristics of carbonates induced by carbonic anhydrase and illustrate their role in sand consolidation.

## 2. Materials

### 2.1. Sampling Methods

This study was conducted on a tropical coral island in the South China Sea characterized by a tropical oceanic monsoon climate. The annual average temperature on the island is 27.4 °C, coupled with an annual mean humidity of 83% and a mean wind speed of 3.4 m/s [28]. The soil composition of tropical coral islands is rich in calcium carbonate, with a fragile and morphologically diverse substrate that is particularly prone to erosion [29]. The vegetation cover on the island is sparsely distributed, with a low extent of coverage. Cyanobacteria-dominated biocrusts account for approximately 6.25% of the total land area [30].

In July 2024, three sample types (bare soil, light biocrusts and dark biocrusts) were collected from the unvegetated soil surface (0–2 cm). Chlorophyll-*a* was used to assess the photosynthetic potential, which serves as an index for the developmental stage of biocrusts. Light biocrusts represent the initial three years of growth, whereas dark biocrusts indicate later stages of more than five years [13,31]. Eight 1 m × 1 m quadrats spaced more than 20 m apart were set up, and five samples were collected from each quadrat. All the samples were stored at 4 °C for enzyme activity tests and bacterial cultivation.

### 2.2. Determination of Chlorophyll-a and Carbonic Anhydrase Activity

The samples were dried at 25 °C and passed through a 35-mesh sieve to remove coarse particles. Chlorophyll-*a* was determined by ethanol spectrophotometry [32]. In the dark environment, 1 g of biocrusts was added to 5 mL of 95% ethanol solution, boiled at 95 °C for 5 min and allowed to cool for 30 min. The absorbance was immediately measured at 665 nm. The carbonic anhydrase activity of biocrusts was determined according to a previous report [33]. The biocrust samples were ground, and then 1 g of each sample was taken and placed into a 15 mL centrifuge tube, after which 1 mL of deionized water was added. The mixture was vortexed for 5 min and centrifuged at 3000× *g* for 5 min at 4 °C. The supernatant was collected for assessment of soil carbonic anhydrase activity. The enzyme activity was measured by the rate of pH reduction by one unit at a temperature of 4 °C. The assay mixture comprised 5 mL of phosphate buffer (20 mmol/L, pH = 8.3), 0.5 mL of either boiled or unboiled sample and 4.5 mL of CO_2_-saturated water. Carbonic anhydrase activity was calculated by the formula U = 10(To/Te − 1). To and Te denote the time of one-unit decrease in pH of boiled or unboiled samples.

### 2.3. Microbiological Analysis

#### 2.3.1. Isolation of Carbonatogenic Bacteria

One gram of soil sample was added to 9 mL of sterile deionized water. After vortexing and standing for 30 min, the sample was diluted and the supernatant was coated onto blue–green 11 (BG-11) and tryptose soybean agar (TSA) medium plates and incubated aerobically at 30 °C. After a period of incubation, single colonies were collected and cultured at 30 °C in sterilized BG-11 or TSB medium. The carbonic anhydrase activity of the strains was determined according to the standard procedure. The strains with carbonic anhydrase activity were stored at −80 °C with 30% (*w/v*) sterilized glycerin.

#### 2.3.2. Bacterial Identification

Bacterial DNA was extracted using EasyPure Bacterial Genomic DNA Kit. The universal primers 27F and 1492R were used to amplify 16S rRNA gene by polymerase chain reaction (PCR). Reaction conditions were as follows: predenaturation at 95 °C for 5 min, 95 °C for 3 min, 57 °C for 30 s and 72 °C for 1 min, for 30 cycles; and a final cycle of 10 min at 70 °C. The PCR products were sequenced using the Sanger method and compared on EZBioCloud website (www.ezbiocloud.net) (accessed on 1 September 2024) to determine the closely related species of the isolated strain. The 16S rRNA gene sequences with the highest similarity in database were taken as references, and the Clustal W method in MEGA-11 (version 11.0) software was used for multiple sequence comparison [34]. Phylogenetic trees depicting the relationships among the related bacteria were constructed using the neighbor-joining method [35].

### 2.4. Sand Consolidation

The strains with high carbonic anhydrase activity were selected, and the method of sand consolidation refers to our previous study [27]. In total, 10 mL of bacterial culture solution, 10 mL of 1 mol/L CaCl_2_ solution and 10 mL of 1 mol/L NaHCO_3_ solution were added to 300 g of quartz sand (with Ca^2+^ removed by 1 mol/L HCl solution and dried early) for seven consecutive days, after the quartz sand was dried. Sterile water was used instead of bacterial solution as the control group (CK). Shear strength was measured using a soil shear testing device (Eijkelkamp, Giesbeek, The Netherlands).

### 2.5. Optimization of Carbonate Formation Conditions

The OD values of bacterial cultures were measured at various temperatures and pH levels to determine the optimal conditions for growth. The concentration of the carbon source (NaHCO_3_) utilized for analyzing carbonic anhydrase-induced carbonate formation ranged from 0.5 to 1.25 mol/L, with an incremental step of 0.25 mol/L. The CaCl_2_ and Ca(C_2_H_3_O_2_)_2_ were selected to test for the optimal calcium source for carbonic anhydrase-induced carbonate formation. The production of carbonic anhydrase-induced calcium carbonate was measured under the optimal condition.

### 2.6. Analysis of the Morphology of Carbonate Crystals

Five grams of particles were scraped from the surface of the consolidated sand for X-ray diffraction (XRD) and scanning electron microscopy (SEM) analysis. The crystal profile of the crystal was analyzed by an X-ray crystal diffractometer (D8 ADVANCE, Berlin, Germany) with CuKα radiation (λ = 0.154 nm), a diffraction angle of 2θ and scanning speeds of 10–80° and 10°/min. The consolidated sand was subjected to ion beam sputtering (ISC150 T, SuPro, Shenzhen, China) and sprayed with gold metal for 60 s. SEM (S-3400 N, Hitachi, Tokyo, Japan) was subsequently used to observe and photograph the cementation effect of carbonate precipitation induced by carbonic anhydrase on sand.

### 2.7. Statistical Analysis

SPSS (version 26.0) was utilized to perform a univariate ANOVA (employing the Tukey HSD test) to assess significant differences in parameter variances. The Clustal W method in MRGA-11 (version 11.0) software was used for multisequence comparison, the predictive tree building model was Tamura-Nei and the neighbor-joining method was adopted for clustering analysis.

## 3. Results

### 3.1. Carbonic Anhydrase Activity and Chlorophyll-a Contents of Biocrusts

**Figure 1:**
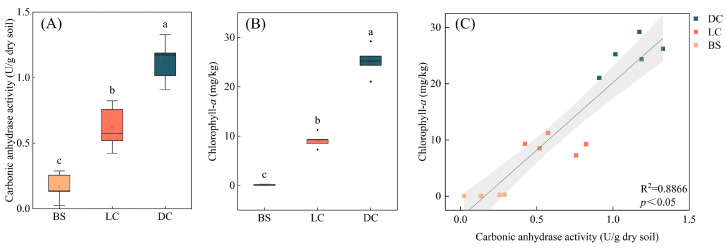
The carbonic anhydrase activity, chlorophyll-*a* content and their correlation. (**A**) Carbonic anhydrase activities of the three sample types. (**B**) Chlorophyll-*a* content in the three sample types. (**C**) Correlation between carbonic anhydrase activity and chlorophyll-*a* content (y = 24.0379x − 3.8156, R^2^ = 0.8866, *p* < 0.05). The presence of distinct letters (a, b, and c) indicates noteworthy variances between groups, with a significance level below *p* < 0.05. The scattered solid shapes depict the specimens obtained from various sites, with yellow, red and blue scatters symbolizing samples collected from bare soil (BS), light biocrusts (LCs) and dark biocrusts (DCs), respectively.

The carbonic anhydrase activities of the three sample types are illustrated in Figure 1A. The carbonic anhydrase activity of the dark biocrusts was 1.20 U/g dry soil, which was significantly higher than those of the light biocrusts and bare soil (*p* < 0.05). The chlorophyll-*a* content in the dark biocrusts was 25.22 mg/kg, which was significantly higher than those of the light biocrusts and bare soil (*p* < 0.05), and that of the dark biocrusts was 2.77 times higher than that of the light biocrusts (Figure 1B). Chlorophyll-*a* serves as an indicator of biocrust development and exhibits a positive correlation with carbonic anhydrase activity (Figure 1C).

### 3.2. Characteristics of Carbonatogenic Bacteria and Their Carbonic Anhydrase Activity

**Figure 2:**
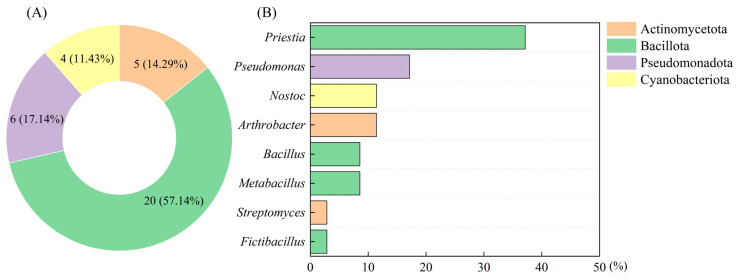
The composition of carbonatogenic bacteria. (**A**) Classification of strains at phylum level. (**B**) Classification of strains at genus level.

**Figure 3:**
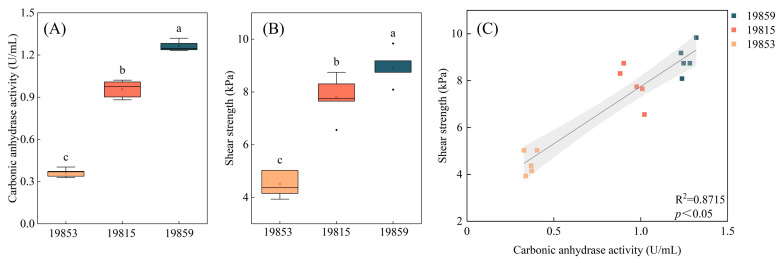
Correlation between carbonic anhydrase activity and shear strength in sand consolidation treated with isolated strains. (**A**) Carbonic anhydrase activity of strain SCSIO19853, SCSIO19815 and SCSIO19859. (**B**) The shear strength in sand consolidation treated with strains SCSIO19853, SCSIO19815 and SCSIO19859. (**C**) Correlation between carbonic anhydrase activity and shear strength in sand consolidation (y = 4.8812x + 2.8698, R^2^ = 0.8715, *p* < 0.05). The presence of distinct letters (a, b, and c) indicates noteworthy variances between groups, with a significance level below *p* < 0.05.

**Figure 4:**
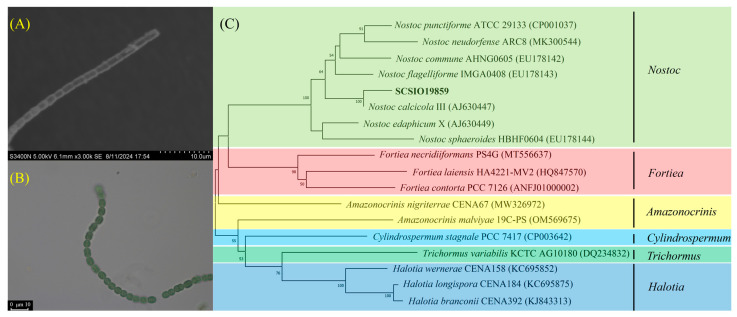
Morphological and phylogenetic tree analyses of strain SCSIO19859. (**A**) Scanning electron microscopy analysis of SCSIO19859. (**B**) Optical microscope analysis of SCSIO19859. (**C**) Phylogenetic tree analysis of SCSIO19859.

In this study, 35 bacterial strains with carbonic anhydrase activity, which belong to four phyla and eight genera, were isolated from biocrusts (Figure 2A,B). The carbonic anhydrase activity of the isolated strains ranged from 0.09 to 1.26 U/mL (Table S1). The correlation between the carbonic anhydrase activity of the strains and the shear strength of the sand consolidation is illustrated in Figure 3A,B. The strain SCSIO19859, which exhibits the highest carbonic anhydrase activity, demonstrated the greatest shear strength in sand consolidation. A significant positive correlation was observed between the microbial carbonic anhydrase activity and the shear strength during sand consolidation (*p* < 0.05) (Figure 3C). Given that strain SCSIO19859 exhibited the highest carbonic anhydrase activity and demonstrated the greatest shear strength in sand consolidation, it was selected for further investigation. The SEM and optical microscope analyses revealed that strain SCSIO19859 exhibited a globular filamentous morphology (Figure 4A,B). Furthermore, the phylogenetic tree analysis indicated that SCSIO19859 could be preliminarily identified as *Nostoc calcicola* (Figure 4C).

### 3.3. Optimal Conditions for Carbonate Formation

**Figure 5:**
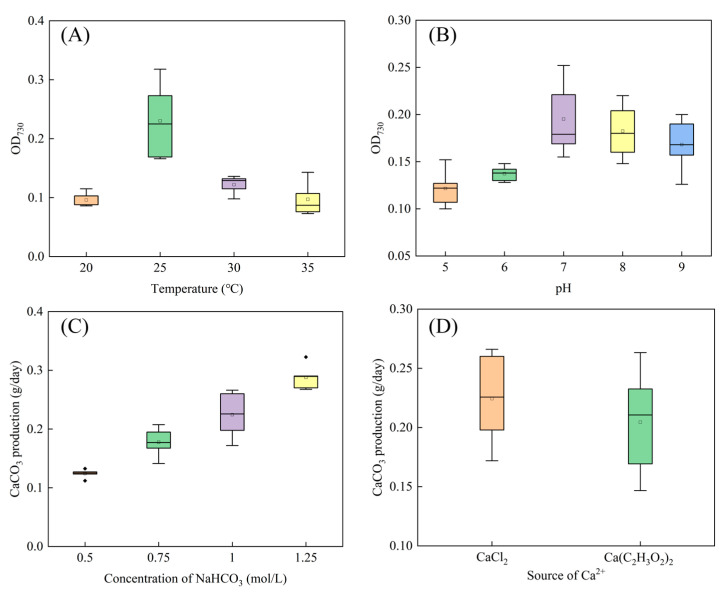
Optimization of carbonate formation conditions of SCSIO19859. (**A**) The optimal temperature for SCSIO19859 growth. (**B**) The optimal pH for SCSIO19859 growth. (**C**) The optimal NaHCO_3_ concentration for CaCO_3_ production. (**D**) The optimal Ca^2+^ source for CaCO_3_ production.

**Figure 6:**
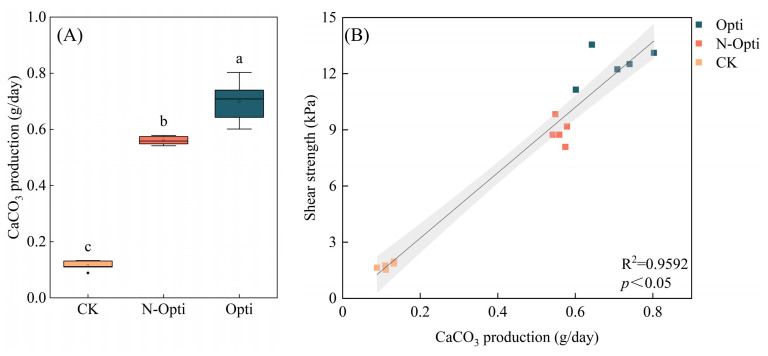
Correlation between CaCO_3_ production and shear strength in sand consolidation obtained through treatment with strain SCSIO19859. (**A**) CaCO_3_ production for optimized (Opti) and non-optimized (N-Opti) conditions, as well as control group (CK). (**B**) Correlation between CaCO_3_ production and shear strength (y = 17.4774x − 0.2793, R^2^ = 0.9592, *p* < 0.05). The presence of distinct letters (a, b, and c) indicates noteworthy variances between groups, with a significance level below *p* < 0.05.

**Figure 7:**
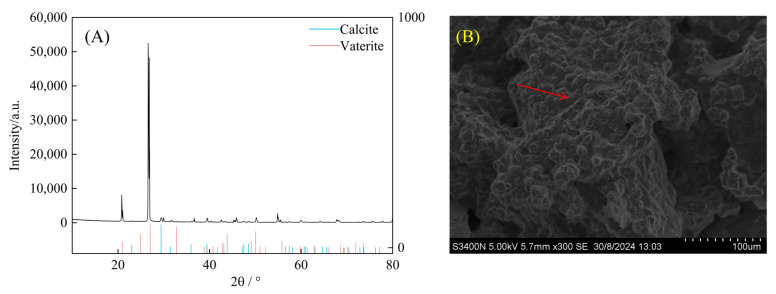
XRD and SEM analyses of calcium carbonate in sand consolidation for treatment with strain SCSIO19859. (**A**) XRD analysis of CaCO_3_ crystals during sand consolidation. The horizontal coordinate in the XRD analysis is twice the incidence angle of the X-ray (2θ), and the vertical coordinate indicates the diffraction intensity. (**B**) SEM analysis of CaCO_3_ crystal during sand consolidation at 300× magnification. The red arrow indicates the presence of calcium carbonate crystals on the surface and within the interstices of sand particles.

As illustrated in Figure 5A,B, the optimal conditions for the growth of strain SCSIO19859 are a temperature of 25 °C and a pH of 7. Under these conditions, strain SCSIO19859 exhibited maximal calcium carbonate production in a 1.5 mol/L NaHCO_3_ solution (Figure 5C). Furthermore, strain SCSIO19859 induced greater calcium carbonate precipitation when CaCl_2_ was used as the calcium source compared to Ca(C_2_H_3_O_2_)_2_ (Figure 5D). Under optimal conditions, strain SCSIO198597 can produce 0.70 g/day of CaCO_3_, a rate that surpasses both that obtained in non-optimized conditions and that of the control group (Figure 6A). Additionally, a significantly positive correlation was observed between CaCO_3_ content and shear strength in sand consolidation (*p* < 0.05) (Figure 7B).

### 3.4. Characteristics of Carbonate Induced by Carbonic Anhydrase

As illustrated in Figure 7A, the XRD analysis revealed that the calcium carbonate particles induced by strain SCSIO19859 primarily consisted of calcite and vaterite. The SEM analysis revealed a significant formation of calcium carbonate crystals produced during the sand consolidation process with the treatment with strain SCSIO19859. These crystals were observed both on the surface and within the interstices between the sand particles (Figure 7B).

## 4. Discussion

### 4.1. Biocrusts Development Promotes the Activity of Microbial Carbonic Anhydrase

The results revealed variations in carbonic anhydrase activity among the different sample types, demonstrating a significant increase concurrent with biocrust formation and development (*p* < 0.05) (Figure 1A). Investigations in karst ecosystems have also revealed variations in the activity of carbonic anhydrase due to distinct karst characteristics, vegetation types and coverage [36]. Similarly, in marine environments, the enzyme’s activity is influenced by variations across different circulation zones and depths [37]. Moreover, soil type also exerts a notable impact on carbonic anhydrase activity, with forests exhibiting the highest levels, followed by Mediterranean ecosystems and deserts, while agricultural soils display the lowest activity [38]. The increase in carbonic anhydrase activity may be attributed to the formation and development of biocrusts. In this study, an increase in chlorophyll-*a* content was observed during the development of biocrusts (Figure 1B). Consistent with our findings, previous studies have also reported a substantial increase in chlorophyll-*a* content with biocrust development [39,40]. Biocrusts exhibiting relatively high levels of chlorophyll-*a* may possess increased photosynthetic capacity, thereby potentially contributing more organic matter to the soil. An increase in soil fertility can provide a greater abundance of resources and energy for the proliferation of microorganisms, thereby facilitating an increase in the microbial population and, ultimately, enhancing the activity of soil microbial carbonic anhydrase [12,41,42]. Moreover, our previous study revealed that the development of biocrusts can also enhance the proliferation of specific microbial communities associated with carbonic anhydrase secretion [27]. The development of biocrusts can also enhance soil physical and chemical properties, including pH, salinity and moisture, thereby promoting an increase in carbonic anhydrase activity [43,44].

Increases in soil carbonic anhydrase activity may contribute to the soil stabilization capabilities of biocrusts. The findings of our previous research indicate a positive correlation between the activity of carbonic anhydrase in biocrusts and their resistance to soil erosion [27]. The strains obtained in this study also revealed a positive correlation between high carbonic anhydrase activity and increased sand-fixation strength (Figure 3C). Previous research has demonstrated that the application of carbonic anhydrase biostimulation significantly enhances the shear strength of soil, resulting in a six-fold increase compared to soil without biostimulation [45]. The compressive strength of concrete treated with carbonic anhydrase-producing bacteria was significantly enhanced [46]. In this study, biocrusts exhibiting well-developed characteristics demonstrated higher carbonic anhydrase activity (Figure 1A), indicating their potential for stronger sand-fixing capabilities. This finding highlights the significance of promoting the development of biocrusts and enhancing carbonic anhydrase activity to improve their ability to stabilize soil for future cultivation and management.

### 4.2. Carbonatogenic Bacteria and Their Role in Sand Consolidation

A total of 35 strains capable of secreting carbonic anhydrase were isolated from biocrusts (Table S1). These strains are classified under the phyla Cyanobacteriota, Actinomycetota, Bacillota and Pseudomonadota (Figure 2A). The findings from our previous investigations demonstrated that these microorganisms constitute the predominant bacterial assemblage within biocrusts [27,30]. The bacteria isolated from biocrusts are also primarily Chloroflexota, Pseudomonadota, Bacteroidota, Actinomycetota and Acidobacteriota [47,48]. It was reported that cyanobacteria possess the ability not only to perform photosynthesis for organic matter synthesis, but also to harbor genes responsible for carbonic anhydrase secretion and regulation, which holds significant potential for calcification [49]. Firmicutes, such as Bacillus, are well known for their high levels of carbonic anhydrase and possess significant potential applications in soil stabilization. Bacillus strains possess the ability to form biofilms, thereby enhancing bacterial colonization, nutrient acquisition and interactions with other microorganisms. Moreover, these strains can produce extracellular polymeric substances, which consist of proteins that facilitate the formation of mineralized products with diverse crystal morphologies, leading to increased inter-particle friction [50,51]. Similarly, Actinomycetota and Pseudomonadota bacteria are also capable of producing carbonic anhydrase, which facilitates CO_2_ sequestration and carbonate generation [52,53]. The carbonic anhydrase activity of the strains obtained in this study ranged from 0.03 to 1.26 U/mL. Among these strains, strain SCSIO19859 demonstrated the highest degree of carbonic anhydrase activity, which is higher than that of the strain *Chryseobacterium gambrini* from the developed karst area of Yunnan Province, but lower than that of the strain *Bacillus* from mine sediment [21,54].

Microbial carbonic anhydrase facilitates the catalysis of carbonate formation, thereby promoting soil stability. The strain SCSIO19859 exhibited enhanced calcium carbonate precipitation when 1.25 mol/L NaHCO_3_ and CaCl_2_ were utilized as substrates (Figure 5). Consistent with other studies, the calcium carbonate particles produced by microbial carbonic anhydrase using calcium chloride as a substrate exhibited higher levels compared to those from other sources of calcium. This can be attributed to the high solubility of the calcium chloride solution and high surface energy [55]. The production of CaCO_3_ was substantially enhanced under optimized conditions compared to non-optimized conditions (*p* < 0.05) (Figure 6A). Moreover, the quantity of CaCO_3_ exhibited a positive correlation with shear strength during the sand consolidation process (Figure 6B). A study conducted along coastlines to induce microbial carbonate precipitation for mitigating coastal erosion also revealed a clear linear relationship between calcium carbonate content and erosion resistance [56]. The XRD analysis revealed that the CaCO_3_ crystals induced by strain SCSIO19859 were predominantly composed of calcite and vaterite (Figure 7A). Carbonic anhydrase is an effective enzyme that catalyzes the formation of calcite, aragonite and precipitates [57]. This process guides newly formed particles into existing calcium spiral structures to facilitate the growth of larger crystals [58]. The stability of calcite crystals derived from calcium carbonate has been reported to be higher than that of aragonite crystals formed by alternative sources of calcium [59]. The strain SCSIO19859 in this study was found to induce calcite production, which could potentially account for the increased formation of calcium carbonate and enhanced sand-fixation strength. The SEM analysis further revealed that calcium carbonate crystals, mediated by carbonic anhydrase, were tightly wrapped around the sand grains (Figure 7B). The process of sand and gravel bonding can be enhanced by the presence of calcium carbonate crystals, which contribute to increased soil density and improved stability [60]. Furthermore, carbonic anhydride crystals can enhance the roughness and friction characteristics of the sand surface, thereby increasing its stability [61]. More crucially, calcium carbonate crystals facilitate robust adhesion among sand particles, thereby enhancing the mechanical stability of the sand [62,63]. In addition to microbial carbonic anhydrase, there may be other factors, including the adsorption of extracellular polymeric substances, the accumulation of organic matter and soil fine particles, or the entanglement of mycelia, which can also enhance soil stability [64,65,66]. Consequently, it is an even greater challenge to explore additional pathways to maintain soil stability during biocrust formation and development.

The present study characterizes the carbonic anhydrase activity of isolated carbonate-producing bacteria and elucidates their role in enhancing soil shear strength during biocrust development. It is plausible that other uncultivated strains also possess carbonic anhydrase activity, which warrants further investigation. Therefore, future research should focus on the isolation and characterization of additional carbonatogenic bacteria. Moreover, efforts should be directed toward optimizing the application of known carbonatogenic bacteria, such as SCSIO19859, to promote biocrust development and enhance soil shear strength under field conditions. Additionally, incorporating an evaluation of the long-term stability of calcium carbonate crystals and exploring potential synergies with other soil stabilization techniques would be of significant importance.

## 5. Conclusions

The present study provides evidence for a link between the carbonic anhydrase activity of carbonatogenic bacteria and the stability of biocrusts. The results demonstrate a significant enhancement in carbonic anhydrase activity with biocrust development (*p* < 0.05). A total of 35 strains capable of secreting carbonic anhydrase were isolated from the biocrusts. The data further revealed a positive correlation between the carbonic anhydrase activity and the shear strength during sand consolidation under these strains. The strain SCSIO19859, which exhibits high carbonic anhydrase activity, can induce the stable formation of calcite and vaterite under optimized conditions. The calcium carbonate crystals can efficiently bind the sand particles, thereby enhancing the shear strength of the sand. This study offers a novel insight into the erosion resistance of biocrusts from the perspective of culturable carbonatogenic bacteria and their carbonic anhydrase. Furthermore, this investigation also highlights the significance of these strains in enhancing soil stability.

## Data Availability

Data is contained within the article or supplementary material. The original contributions presented in this study are included in the article/supplementary material. Further inquiries can be directed to the corresponding author.

## Supplementary Materials

The following supporting information can be downloaded at: https://www.mdpi.com/article/10.3390/microorganisms13030523/s1, Table S1: The carbonic anhydrase activity and closely related species of isolated strains.
