# Supplementary material for: Characteristics of Carbonatogenic Bacteria and Their Role in Enhancing the Stability of Biocrusts in Tropical Coral Islands
Source: Microorganisms. 2025 Feb 27;13(3):523. doi: 10.3390/microorganisms13030523 (PMC11945846; doi:10.3390/microorganisms13030523)
Supplement: Supplementary file 1 [file microorganisms-13-00523-s001.zip › microorganisms-3456230-supplementary.pdf]

**Table S1**. The carbonic anhydrase activity and closely related species of isolated strains. Abbreviations: Carbonic anhydrase activity (CA).

| Strain number | CA (U/mL) | Closely related species | Similarity (%) |
|---|---|---|---|
| SCSIO19816 | 0.29 | *Arthrobacter nitrophenolicus* AOFD01000111 | 100.00 |
| SCSIO19817 | 0.59 | *Arthrobacter globiformis* BAEG01000072 | 98.78 |
| SCSIO19818 | 0.41 | *Arthrobacter ipis* JAAOXD010000003 | 98.64 |
| SCSIO19819 | 0.27 | *Arthrobacter pokkalii* KM507333 | 98.56 |
| SCSIO19821 | 0.21 | *Streptomyces globosus* (AJ781330) | 99.30 |
| SCSIO19823 | 0.77 | *Bacillus aranthracis* MACE01000012 | 99.93 |
| SCSIO19825 | 0.03 | *Bacillus albus* MAOE01000087 | 99.93 |
| SCSIO19815 | 0.96 | *Bacillus cereus* AE016877 | 100.00 |
| SCSIO19828 | 0.21 | *Fictibacillus phosphorivorans* JX258924 | 100.00 |
| SCSIO19829 | 0.40 | *Metabacillus indicus* JGVU01000003 | 99.86 |
| SCSIO19831 | 0.87 | *Metabacillus indicus* JGVU01000003 | 99.78 |
| SCSIO19832 | 0.33 | *Metabacillus idriensis* AY904033 | 98.15 |
| SCSIO19833 | 0.50 | *Priestia flexa* BCVD01000224 | 99.86 |
| SCSIO19834 | 0.64 | *Priestia megaterium* JJMH01000057 | 98.81 |
| SCSIO19835 | 0.37 | *Priestia megaterium* JJMH01000057 | 99.79 |

**Continued Table S1**. The carbonic anhydrase activity and closely related species of isolated strains.

| Strain number | CA (U/mL) | Closely related species | Similarity (%) |
|---|---|---|---|
| SCSIO19836 | 0.09 | *Priestia aryabhattai* EF114313 | 99.79 |
| SCSIO19837 | 0.84 | *Priestia flexa* BCVD01000224 | 99.86 |
| SCSIO19839 | 0.32 | *Priestia megaterium* JJMH01000057 | 98.81 |
| SCSIO19841 | 0.65 | *Priestia paraflexa* FN999943 | 98.70 |
| SCSIO19842 | 0.74 | *Priestia aryabhattai* EF114313 | 98.67 |
| SCSIO19844 | 0.22 | *Priestia qingshengii* JX293295 | 96.97 |
| SCSIO19845 | 0.16 | *Priestia flexa* BCVD01000224 | 99.86 |
| SCSIO19846 | 0.29 | *Priestiaa megaterium* JJMH01000057 | 98.81 |
| SCSIO19847 | 0.26 | *Priestia paraflexa* FN999943 | 98.70 |
| SCSIO19848 | 0.33 | *Priestia aryabhattai* EF114313 | 98.67 |
| SCSIO19850 | 0.89 | *Pseudomonassolani* LC744517 | 99.93 |
| SCSIO19851 | 0.66 | *Pseudomonas alcaligenes* BATI01000076 | 99.22 |
| SCSIO19852 | 0.53 | *Pseudomonas otitidis* AY953147 | 98.64 |
| SCSIO19853 | 0.36 | *Pseudomonas tohonis* LC645211 | 99.93 |
| SCSIO19854 | 0.80 | *Pseudomonas solani* LC744517 | 99.93 |
| SCSIO19856 | 0.75 | *Pseudomonas alcaligenes* BATI01000076 | 99.22 |
| SCSIO19859 | 1.26 | *Nostoc edaphicum* AJ630449 | 98.36 |
| SCSIO19803 | 0.41 | *Nostoc punctiforme* CP001037 | 98.22 |
| SCSIO19802 | 0.32 | *Nostoc flagelliforme* EU178143 | 98.14 |
| SCSIO19857 | 0.36 | *Nostoc calcicola* AJ630447 | 99.57 |
